# Estimation of the Incidence of Congenital Syphilis in Mexico Between 2019 and 2023

**DOI:** 10.7759/cureus.63913

**Published:** 2024-07-05

**Authors:** Erick A Rochel-Perez, Mario A Martin-Dorantes, Nina Mendez-Dominguez

**Affiliations:** 1 Departamento de Investigación, Hospital Regional de Alta Especialidad de la Península de Yucatán-IMMS BIENESTAR, Mérida, MEX; 2 Departamento de Investigación, Hospital Regional de Alta Especialidad de la Península de Yucatán-IMSS BIENESTAR, Mérida, MEX; 3 Research and Learning, Hospital Regional de Alta Especialidad de la Peninsula de Yucatan-IMSS BIENESTAR, Mérida, MEX

**Keywords:** treponema pallidum, live births, vulnerable population, incidence, congenital syphilis

## Abstract

Introduction

Congenital Syphilis (CS) is considered the second leading cause of preventable death in developing countries. The last report of the incidence rate of CS was made in 2017.

Objective

The objective of the study was to estimate the incidence of CS between 2019-2023.

Materials and methods

This is a retrospective study for which data were obtained from the new cases of CS reported in the *Epidemiological Bulletin *for 2019-2023 and from the newborn population records reported in the National Institute of Statistics and Geography and the National Population Council.

Results

In Mexico, the incidence rate of CS is 3.20 per 10,000 births. An increment of CS cases was observed between 2019-2023, with a higher number of cases in 2022.

Conclusion

A democratization of detection and prompt management is needed to reduce transmission, particularly among the most vulnerable population.

## Introduction

Congenital syphilis (CS) is caused by the transmission of the bacterium *Treponema pallidum* (*T. pallidum*) from an infected mother to the newborn; this disease is one of the main preventable causes of late fetal death due to maternal-fetal transmission in developing countries [1]. In addition to fetal death, it can lead to premature birth, spontaneous abortion, perinatal death, non-immune hydrops, and congenital syphilis syndromes. These syndromes are classified as early congenital syphilis when clinical manifestations appear within the first 2 years of age, and late congenital syphilis when clinical manifestations appear after 2 years of age [2].

The incidence of CS is complex to obtain due to factors such as the lack of commitment to prenatal care and the limited access to and availability of diagnostic and screening tests for this disease. Cuba, Thailand, and the Dominican Republic are some of the countries that have received validation from the World Health Organization (WHO) for having dropped mother-to-child transmission of syphilis; however, there are regions in the world where CS persists. The African region is one of the most affected, with an estimated 404,000 cases in 2016, representing 57% of the cases worldwide in that same year. Even developed countries like the United States have not only failed to eradicate this disease, but its incidence is also increasing, with 918 cases in 2017 and 2,148 cases in 2020 [3,4].

The incidence of syphilis has increased among Mexican women aged 15 to 24 years. The presence of this disease in women of reproductive age, mainly between 20 and 24 years old, has favored the increase in CS cases. Herrera-Ortiz and collaborators reported that in Mexico there was an increase in CS cases of 181% between 2013 and 2017 and of 307% between 2017 and 2019 [5,6]. However, there is no updated literature reporting new cases of CS in Mexico in recent years. Therefore, the aim of this study is to estimate the incidence of congenital syphilis in Mexico between 2019-2023, which would allow observing the continuous presence of this disease in the country and its increasing incidence.

## Materials and methods

The present study is a retrospective cross-sectional descriptive observational study; it included the report of the number of pediatric patients diagnosed and registered as new cases of CS in a health institution in Mexico and included as part of the weekly report in the *Epidemiological Bulletin *of the National Epidemiological Surveillance System of Mexico as new cases of CS coded with A50, based on the tenth edition of the International Statistical Classification of Diseases and Related Health Problems (ICD-10) [7].

CS cases were obtained from the reports published by the *Epidemiological Bulletin *for the last week of the years 2019, 2020, 2021, 2022, and 2023 to include all cases registered in each year. The number of cases reported in the *Epidemiological Bulletin *in each federal entity was considered to identify the regions with the most reported cases in the country. These data were compared with birth records from the 32 federal entities as reported by the National Institute of Statistics and Geography (INEGI) in the years 2019-2022 and the birth projections in each federal entity made by the National Population Council (CONAPO) in 2023 [8,9].

Using Microsoft Excel (Microsoft Corporation, Redmond, USA) the incidence rates in the federal entities were calculated for each selected year, using the records of new CS cases reported in the *Epidemiological Bulletin *as the numerator and the births registered by INEGI in 2019-2022 and the birth projection in 2023 as the denominator. The results were multiplied by 10,000 to obtain the corresponding rates. Additionally, the accumulated rate for the five years of the study was calculated using the sum of new CS cases reported by the *Epidemiological Bulletin *and the sum of birth records reported by INEGI and CONAPO as numerators and denominators, respectively [10]. The data obtained were processed using the same software to generate maps depicting the intensity of cumulative incidence for each year.

No prospective measures were included in the present study; a compilation of secondary, anonymized, and open-access data from medical records present at http://www.dgis.salud.gob.mx/contenidos/sinais/subsistema1.html was conducted. The authors adhered to the guidelines of the Helsinki Declaration for the use of biobanks (https://www.wma.net/es/policies-post/declaracion-de-helsinki-de-la-amm-principios-eticos-para-las-investigaciones-medicas-en-seres-humanos/).

## Results

The cumulative incidence rate of CS was 3.20 per 10,000 live births during the period 2019-2023. There was an increase in the number of cases between 2019 and 2020, with 460 (2.19 per 10,000 live births) and 493 (3.02 per 10,000 live births), respectively. In 2021, the cases decreased to 482 (2.52 per 10,000 live births); however, they increased in 2022 and 2023, with 825 (4.41 per 10,000 live births) and 813 (3.93 per 10,000 live births), respectively (Table 1).

**Table 1:** Distribution of incidence rate per 10,000 live births at the national level, 2019-2023.

Entity	Rates per 10,000 live births					
	2019	2020	2021	2022	2023	Cumulative
Aguascalientes	4.03	7.43	13.1	16.62	4.94	8.07
Baja California	19.67	19.52	13.98	18.19	16.61	17.53
Baja California Sur	20.53	14.81	1.95	8.08	1.56	9.29
Campeche	0	3.77	0	2.17	3.78	1.79
Coahuila	3.54	0.85	3.55	9.84	7.1	5
Colima	17.24	30.48	28.40	22.51	29.76	25.49
Chiapas	0.91	0.3	0.06	0.06	0	0.26
Chihuahua	0.66	1.04	4.34	10.25	8.81	5.02
Ciudad de México	0.67	0.3	0.10	1.11	1.48	0.76
Durango	4.25	1.79	0.64	0.99	1.36	1.85
Guanajuato	0.81	1.3	2.99	4.18	1.67	2.19
Guerrero	0.63	0.66	0.14	0.41	0.74	0.52
Hidalgo	0.67	0.26	0.50	0	0.22	0.34
Jalisco	3.39	3.66	7.11	8.06	7.13	5.88
México	0.23	0.16	0.04	0.64	0.43	0.31
Michoacán	0.97	0.67	1.44	3.47	3.04	1.94
Morelos	0.66	0.4	0.77	0	0	0.36
Nayarit	2.63	1.8	7.14	17.69	7.33	7.55
Nuevo León	1.1	4.45	7.22	8.65	10.16	6.42
Oaxaca	0	0	0	0	0.12	0.02
Puebla	0.31	0.56	0.76	2	0.3	0.76
Querétaro	0.26	0.3	0	1.53	0.24	0.45
Quintana Roo	0.65	2.5	0.38	0	0	0.63
San Luis Potosí	1.26	2.6	0.22	11.95	12.13	5.72
Sinaloa	9.47	17.42	3.58	4.67	8.09	8.40
Sonora	7.06	29.35	2.87	14.31	23.1	14.55
Tabasco	2.27	0.34	0.23	0	0	0.61
Tamaulipas	2.14	2.68	6.04	10.39	8.21	5.88
Tlaxcala	0	0	0	0.52	0	0.09
Veracruz	1.53	1.42	0.38	1.1	1.63	1.22
Yucatán	2.09	0.34	1.78	5.01	3.25	2.49
Zacatecas	0.31	0.75	1.05	3.34	1.58	1.37
Total	2.19	3.02	2.52	4.41	3.93	3.20

**Figure 1:**
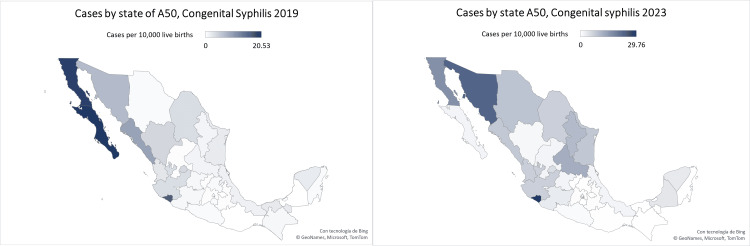
Comparison of the geographic distribution of congenital syphilis cases between 2019 and 2023. The data used in this figure was obtained from the *Epidemiological Bulletin of Mexico*, which is part of the open data present in this link: https://www.gob.mx/salud/acciones-y-programas/direccion-general-de-epidemiologia-boletin-epidemiologico

The geographical distribution of CS cases was heterogeneous, with higher incidence in the northern region of the country. Aguascalientes, Baja California, Baja California Sur, Colima, and Sonora showed the highest incidence rates. The states located in the central and southern regions of the country had lower incidence rates, including Oaxaca, Chiapas, State of Mexico, Tlaxcala, and Hidalgo (Figure 1).

## Discussion

Congenital syphilis (CS) is a disease that continues to affect newborns worldwide, despite the extensive knowledge of the disease, treatment, and optimal preventive measures. According to the Pan American Health Organization (PAHO) in 38 countries of the region the incidence rate of CS has been increasing from 0.3 cases per 1,000 live births in 2009 to 0.61 cases per 1,000 live births in 2020. Likewise, the incidence of CS in the United States has increased throughout the 21st century, especially in states like Texas and California. The results of our study show a similar trend, with a progressive increase in the incidence of this disease during the study period, with federal entities representing the highest number of cases nationwide. This pattern is also seen in previous studies realized in Mexico like the study of García-Cisneros and collaborators which reported that the cases of CS increased from 62 cases in 2010 to 372 cases in 2019 [5,11,12].

Brazil is one of the countries in the Americas with a high incidence, with an increase in CS cases from 6,949 cases in 2010 to 24,493 cases in 2017 [3]. This increase in CS cases is related to limited access to healthcare services, late initiation of prenatal care, low socioeconomic status, geographic location, and limited diagnostic tests for syphilis [13,14]. Similarly to Brazil, the increase in CS cases in Mexico could be due to limited access to healthcare services for the population living in rural or marginalized areas. It is estimated that 48.8% of the Mexican population does not have social security, while 17.2% of pregnant women report insufficient prenatal care, and 4.2% of women are not attended to during childbirth by medical personnel [15,16].

In 2010, the PAHO set a goal to reduce the incidence of CS to <5 cases per 10,000 live births by 2015. Based on the results of our study, Mexico achieved this goal by presenting a cumulative incidence rate of 3.20 cases per 10,000 births between 2019 and 2023 [17]. To achieve these results, the Mexican Official Standard (NOM) NOM-007-SSA2-2016 for prenatal care was established, which indicates that the initial tests for syphilis and HIV should be offered in the first trimester and before childbirth for timely diagnosis [17]. However, strategies for preventing this disease and promoting adherence to prenatal care among women of reproductive age need to be established, as the increase from 43 cases in 2013 to 825 cases in 2023 indicates that it is still necessary to ensure compliance with this NOM and access to adequate treatment for the entire population [5].

A limitation of this study is that the information was obtained from publicly accessible databases, which are prone to underreporting or miscoding, potentially affecting the accuracy of our analysis. The calculated incidence in our study demonstrates geographical heterogeneity; however, there is likely underdiagnosis in vulnerable populations, particularly in states like Oaxaca and Chiapas, which recorded the lowest national incidence. This underdiagnosis is likely associated with limited access to healthcare services, unfavorable socioeconomic conditions, cultural barriers, and limited awareness of the importance of health promotion measures [18]. Women who have timely and adequate access to prenatal care typically exhibit characteristics such as higher educational levels, urban residence, higher socioeconomic status, lower marginalization index, and fewer pregnancies [16,19].

## Conclusions

To eradicate cases of CS, it is essential to establish collaboration among the government, health organizations, and Mexican society through specific strategies. Implementing awareness campaigns that promote adequate prenatal care and provide clear information about CS screening tests is fundamental. Additionally, improving access to healthcare services, particularly in marginalized communities, is crucial.

It is imperative to promote diagnostic tests as a mandatory component of prenatal care and to enhance diagnostic testing sites in states with high incidence for timely detection. In conclusion, CS is a condition that remains neglected in terms of prevention, diagnosis, and timely management in Mexico. Addressing the needs of the most vulnerable populations in the context of CS prevention, diagnosis, and management is essential. This necessitates implementing measures to improve access to healthcare during prenatal care, promoting health education, and addressing the cultural and socioeconomic barriers faced by these communities.
